# Increased Serum Levels of Phoenixin-14, Nesfatin-1 and Dopamine Are Associated with Positive Pregnancy Rate after Ovarian Stimulation

**DOI:** 10.3390/jcm12226991

**Published:** 2023-11-08

**Authors:** Magdalena Piróg, Robert Jach, Michał Ząbczyk, Joanna Natorska

**Affiliations:** 1 Gynecological Endocrinology Department, Jagiellonian University Medical College, 31-501 Krakow, Poland; robert.jach@uj.edu.pl; 2 Department of Thromboembolic Disorders, Institute of Cardiology, Jagiellonian University Medical College, 31-202 Krakow, Poland; michal.zabczyk@uj.edu.pl (M.Z.); j.natorska@szpitaljp2.krakow.pl (J.N.); 3 Krakow Centre for Medical Research and Technologies, St. John Paul II Hospital, 31-202 Krakow, Poland

**Keywords:** reproductive medicine, reproductive endocrinology, infertility nesfatin-1, phoenixin-14, ovarian stimulation

## Abstract

Background: We study the relationship between phoenixin (PNX-14), nesfatin-1 (NES-1), dopamine (DA) and oxytocin (OT) levels together with pregnancy rates in women after ovarian stimulation (OS). Methods: In a prospective case–control study, 56 infertile women were enrolled from the Department of Gynecological Endocrinology University Hospital. Infertile women age < 40 years old, with polycystic ovary syndrome (PCOS), confirmed tubal patency and suitable sperm quality were included. Blood samples were drawn twice—before the initiation of OS and before the human chorionic gonadotropin (hCG) administration. Assessments of PNX-14, NES-1, DA and OT serum levels were performed. Pregnancy rates after OS were observed. Results: Pregnant women showed higher baseline NES-1 and OT levels (+29.2% and +44%) but not PNX-14 and DA levels when compared to non-pregnant ones. In pregnant women, positive correlations between OT and prolactin, PRL (r = 0.47, *p* = 0.04), as well as between OT and NES-1 (r = 0.55, *p* = 0.02), were observed at baseline. At baseline, an OT level increase was associated with a positive pregnancy rate (per 100 pg/mL, OR = 1.39, 95% CI 1.04–1.74), while after OS, higher PNX-14 was a predictor of pregnancy (by 10 pg/mL, OR = 1.23, 95%CI 1.07–1.39). Post-stimulation PNX-14, NES-1 and DA concentrations were higher in pregnant women compared to non-pregnant ones (+17.4%, +26.1%, and +45.5%, respectively; all *p* < 0.05). In the pregnant group, OT levels were 2.7-times lower than in the remainder (*p* = 0.03). Moreover, in pregnant participants, a negative association between NES-1 and PNX (r = −0.53, *p* = 0.024) was observed. Conclusion: Elevated PNX-14, NES-1 and DA along with decreased OT levels were observed in women who achieved pregnancy.

## 1. Introduction

Ovulatory disorders are the major causes of female infertility and account for approximately 30–40% of women and around 20% of all infertility problems in couples [1]. Regular ovulatory cycles confirm a proper functioning of the hypothalamic–pituitary–ovarian (HPO) axis. The proper pulsatile release of hypothalamic gonadotropin-releasing hormone (GnRH) regulates pituitary gonadotropins secretion—luteinizing hormone (LH) and follicle-stimulating hormone (FSH)—which in turn condition ovarian excretion of estrogen (E_2_) and progesterone (P_4_) [2]. However, it has been suggested that particular neuropeptides may modulate the HPO function. Phoenixin (PNX) and nesfatin-1 (NES-1), endogenous neuropeptides localized in the hypothalamus, are thought to regulate the reproductive function by influencing the HPO axis [3]. In vitro studies have shown that PNX-14 enhances GnRH receptor expression and, consequently, up-regulates the secretion of FSH and LH [4]. Moreover, PNX-14 may simulate follicular cell development by accelerating the proliferation of human granulosa cells and inducing E_2_ secretion [5]. Interestingly, an increased PNX-14 concentration was observed in women with polycystic ovary syndrome (PCOS), the most common ovulatory disorder [6]. NES-1 acts directly on the pituitary gland and it has positive effects on reproductive hormones. Reproductive hormones, instead, modulate NES-1 expression in the hypothalamus and pituitary cells [7]. In rats, a high co-expression of NES-1 and PNX-14 in the central nervous system has been proven [8,9]. Moreover, NES-1 is co-expressed with the HPO axis hormones, including oxytocin (OT) [10]. OT is known to modulate sexual behavior, labor and lactation, but its role in conceiving is unknown [10]. Both OT and dopamine (DA) are also suggested to modulate the HPO function [11,12]. DA is a catecholamine neurotransmitter found in the brain and peripheral organs [13]. DA exerts an inhibitory effect on prolactin release. Available studies have revealed a positive association between PCOS, low DA and hyperprolactinemia [14]. Elevated DA levels decrease the prolactin concentration. Hyperprolactinemia, consequently, exerts an inhibitory effect on the gonadotrophins [15]. Both OT and DA are also able to reduce the severity of conditions related to hyperprolactinemia such as fear, anxiety and depression [16,17], and thus, it might facilitate pregnancy rates after ovarian stimulation (OS). 

Dysfunction of the HPO axis contributes to anovulatory cycles and OS is a treatment of choice. OS induces the development of at least one mature follicle in 72–80% of women, but only 40–47% of them conceive. Taken together, the effectiveness of OS depends on two stages: an adequate response of the HPO axis to OS, together with undisturbed embryonic implantation and development. Therefore, both of the stages might be associated with the concentration of neuropeptides, such as PNX-14, NES-1, OT and DA.

To the best of our knowledge, serum PNX-14 and NES-1 levels and their potential associations with DA and OT in pregnant women after OS have not been previously investigated. Therefore, this study aimed to evaluate whether PNX-14 and NES-1 as well as DA and OT are affected by OS and associated with pregnancy rates. 

## 2. Materials and Methods

### 2.1. Study Design

This prospective, case–control, single-center study was conducted between January 2022 and June 2023 in the Department of Gynecological Endocrinology and Gynecology, Jagiellonian University Medical College, Krakow, Poland. 

### 2.2. Ethics

The Ethics Committee at Jagiellonian University Medical College approved the study (reference number KBET 1072.6120.220.2021 from 29 September 2021), and participants provided informed consent as per the Declaration of Helsinki. The study was registered at www.clinicaltrials.gov (NCT04166825), accessed on 27 July 2023 and is reported according to the CONSORT guidelines.

### 2.3. Study Participants

A total of 56 infertile women with polycystic ovary syndrome were enrolled.

The inclusion criteria included [18,19,20] the following:
  1. Age < 40 years old.
  2. Infertility diagnosed as the failure to conceive after 6 months (for women < 40 years old) or one year (for women <35 years old) of regular intercourse without contraception.
  3. PCOS was diagnosed based on the Rotterdam PCOS Diagnostic Criteria (at least two of oligo- or anovulation, clinical and/or biochemical hyperandrogenism, or polycystic ovaries on ultrasound), after the exclusion of related disorders.
  4. Excluded fallopian tube obstruction by hysterosalpingography.
  5. Confirmed suitable sperm quality (per WHO 2021 guidelines).
The exclusion criteria included the following:
  - Poor ovarian response (POR): (1) advanced maternal age ≥ 40 years or any other risk factor for POR; (2) an abnormal ovarian reserve test (i.e., antral follicle count; AFC < 5–7 follicles or anti-Müllerian hormone levels; AMH < 0.5–1.1 ng/mL).
  - Use of hormonal therapy within 3 months before OS: oral contraceptives or dienogest therapy (due to endometriosis).
  - Concomitant diseases: severe hypertension, diabetes mellitus, known malignancy, any chronic inflammatory diseases (e.g., rheumatoid arthritis) or signs of an acute infection, advanced chronic renal disease (estimated glomerular filtration rate (eGFR) < 30 mL/min), or international normalized ratio (INR) more than 1.2 at the day of blood draw.
  - Endometriosis stages III or more according to the revised American Society for Reproductive Medicine score (rASRM).

We collected data on risk factors and comorbidities. Arterial hypertension was defined as systolic blood pressure ≥ 140 mmHg and/or diastolic blood pressure ≥ 90 mmHg or taking antihypertensive medication. Obesity is defined as having a body mass index (BMI) ≥ 30 kg/m^2^. Diabetes mellitus was defined in accordance with the American Diabetic Association Criteria [21]. 

### 2.4. Ovarian Stimulation

An ovarian stimulation protocol initiation was preceded by a lack of any growing follicle (greater than 10 mm in diameter) by transvaginal ultrasound (TVU). None of the women used oral contraceptives or estradiol priming for cycle initiation. 

All women received letrozole orally (2.5 mg daily) from day 3 of the menstrual cycle for 5 days. TVU examinations were performed every second day from day 10 of the cycle to monitor the number and size of developing follicles along with endometrial thickness [19]. If at least one follicle (not more than 3) has achieved ≥18 mm and endometrial thickness attained ≥7 mm, the patient received 250 mcg human chorionic gonadotropin (hCG; Ovitrelle, Merck Europe B. V., Darmstadt, Germany) s.c. to induce ovarian ovulation. If menstruation did not occur after 14 days from hCG administration serum, βhCG was performed for diagnosis of pregnancy. The evolution of the pregnancy was not recorded.

### 2.5. Blood Sampling and Analyses

Venous blood samples were drawn with minimal stasis using atraumatic venipuncture at 08.00–10.00 AM, after an overnight fast and a 10 min rest twice, at study entry and before hCG administration. FSH, LH, estradiol, thyroid-stimulating hormone (TSH) and prolactin (PRL) were assayed using routine laboratory techniques. Serum AMH levels were measured using the enzyme-linked immunosorbent assay kit (Beckman Coulter Generation II ELISA assay, Marseilles, France). 

Serum OT, DA (Abcam, Cambridge, UK), NES-1 (ThermoFisher Scientific, Waltham, MA, USA) and PNX-14 (FineTest Biotech Inc., Boulder, CO, USA) were assayed using ELISA according to manufacturers’ instructions. All measurements were performed by technicians blinded to the origin of the samples. Intra-assay and inter-assay coefficients of variation were 5–7%. 

### 2.6. Data Management and Statistical Analyses

Statistical analysis was performed with the STATISTICA 13.0 software (StatSoft, Kraków, Poland). 

Categorical variables are presented as numbers and percentages. Continuous variables are expressed as mean ± standard deviation or median and interquartile range (IQR), as appropriate. The Kolmogorov–Smirnov test was used to assess conformity with a normal distribution, whereas non-normally distributed data were analyzed using Kruskal–Wallis followed by Mann–Whitney’s comparison tests. For paired data, the Student’s *t* test or the Wilcoxon signed-rank tests were used as appropriate. Categorical variables were analyzed using either the chi^2^ test or Fisher’s exact test. Pearson’s correlation coefficient (Pearson’s r) or Spearman’s rank correlation coefficient were calculated to assess the linear correlations between variables with a normal or non-normal distribution, respectively. Univariable and multivariable logistic regression models were performed to identify clinical and laboratory factors associated with pregnancy. The multivariable model was fitted using the backward stepwise regression. Variables that were associated with pregnancy with a significance level of *p* < 0.2 in the bivariate models were selected for possible inclusion in the multivariable logistic regression models. Associations between the variables were expressed as odds ratios with 95% confidence intervals. The study was powered to have a 90% chance of detecting a 10% difference in PNX-14, using a *p*-value of 0.05. Based on previous data [6], to demonstrate such a difference or greater, at least 11 patients were required in each group. The comparison of the studied groups using the Student’s *t*-test for unrelated variables showed that at the significance level of *p* = 0.05, it allows us to detect differences equal to 0.86 of standard deviation between the means with a probability of 90%. Two-sided *p*-values < 0.05 were considered statistically significant. 

## 3. Results

### 3.1. Participants’ Characteristics

Basic characteristics of the investigated subjects along with their hormonal profiles are presented in Table 1. Ovulation was noted in 44 (78.6%) women. The pregnancy rate after OS was 32.1%. There were no intergroup differences in age, BMI or duration of infertility among the two groups. In the pregnant group, elevated pre- and post-treatment estradiol levels were observed (+12.5% and +13.7%, respectively) compared to the non-pregnant group (Table 1). No further differences in hormonal profiles at the initiation of the treatment and after OS were detected (Table 1).

### 3.2. Neuropeptides before OS

Serum levels of neuropeptides are presented in Table 2. In the whole cohort of patients, OT levels were weakly associated with BMI (r = 0.26, *p* = 0.048). We also observed a positive correlation between OT and FSH (r = 0.47, *p* = 0.0002). There were no other associations between neuropeptides and clinical or laboratory parameters (all *p* > 0.05).

Pregnant women showed higher baseline NES-1 and OT levels (+29.2% and +44%) when compared to non-pregnant ones (Table 2). Among pregnant women, positive correlations were observed between baseline OT and PRL (r = 0.47; *p* = 0.04), as well as OT and NES-1 (r = 0.55; *p* = 0.02). The OT level increase was associated with positive pregnancy rates both in the univariable (per 100 pg/mL, OR = 1.43, 95% CI 1.09–1.76) and multivariable logistic regression analysis (per 100 pg/mL, OR = 1.39, 95% CI 1.04–1.74), while pregnancy was not predicted by other neuropeptides measured on admission.

### 3.3. Post-Stimulation Changes in Neuropeptide Levels 

Serum levels of neuropeptides after OS are presented in Table 2. In the whole cohort, OS was associated with an increase in the NES-1 level (+15%, *p* = 0.0019), but no changes were observed for the other neuropeptides (all *p* > 0.05). Similarly, we found no differences in all assessed neuropeptides concerning ovulation (all *p* > 0.05). In the whole cohort of patients, a positive correlation between DA and PRL was observed (r = 0.32, *p* = 0.02), while NES-1 was positively associated with LH (r = 0.31, *p* = 0.021).

Post-stimulation PNX-14, NES-1 and DA concentrations were higher in pregnant women compared to non-pregnant ones (+17.4%, +26.1% and +45.5%, respectively) (Table 2). In the pregnant group, OT levels were 2.7-times lower than in the remaining one. 

**Figure 1:**
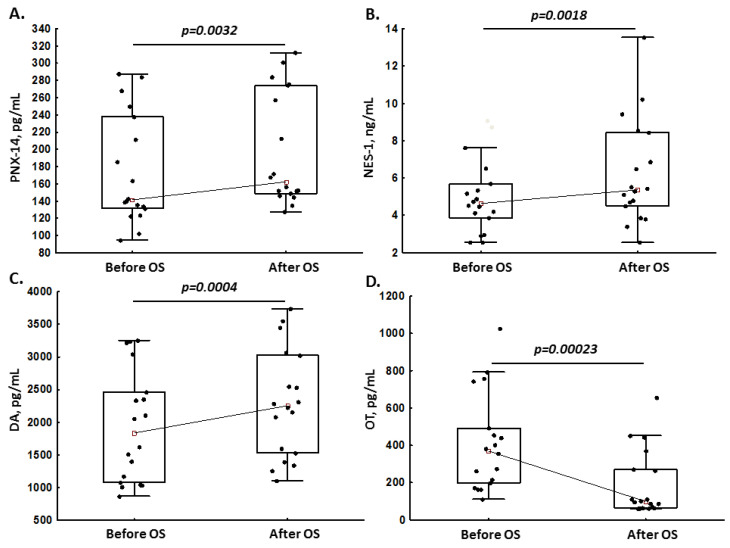
Phoenixin-14 (PNX-14; panel (**A**)), nesfatin-1 (NES-1; panel (**B**)), dopamine (DA; panel (**C**)) and oxytocin (OT; panel (**D**)) levels in pregnant women before and after ovarian stimulation (OS).

Compared to baseline, in pregnant women, levels of PNX-14 (+14.8%; Figure 1A), NES-1 (+15.7%; Figure 1B) and DA (+22.8%; Figure 1C) increased after OS, while OT decreased by 73% after OS (Figure 1D). In non-pregnant women, the median levels of all neuropeptides remained unchanged after OS (all *p* > 0.05, Table 2). 

**Figure 2:**
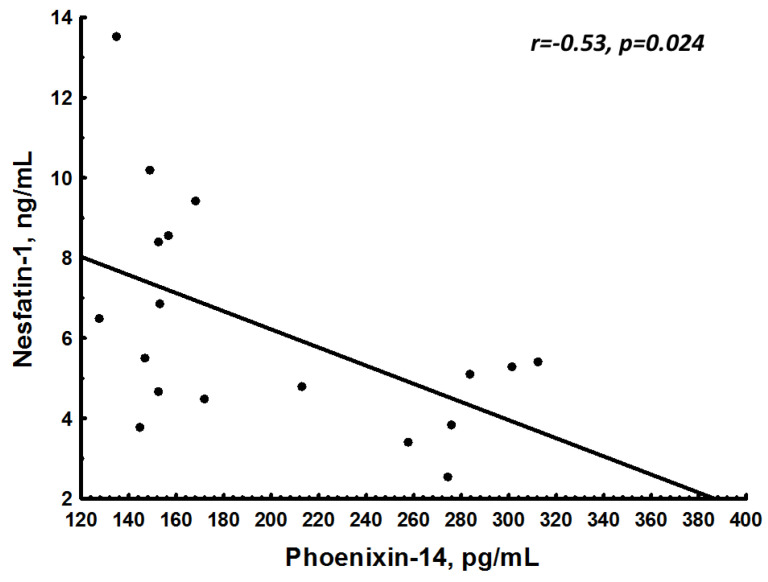
Association between nesfatin-1 (NES-1) and phoenixin-14 (PNX-14) in pregnant women.

Solely in pregnant women, a negative association between NES-1 and PNX-14 (r = −0.53; *p* = 0.024) was observed (Figure 2). 

**Figure 3:**
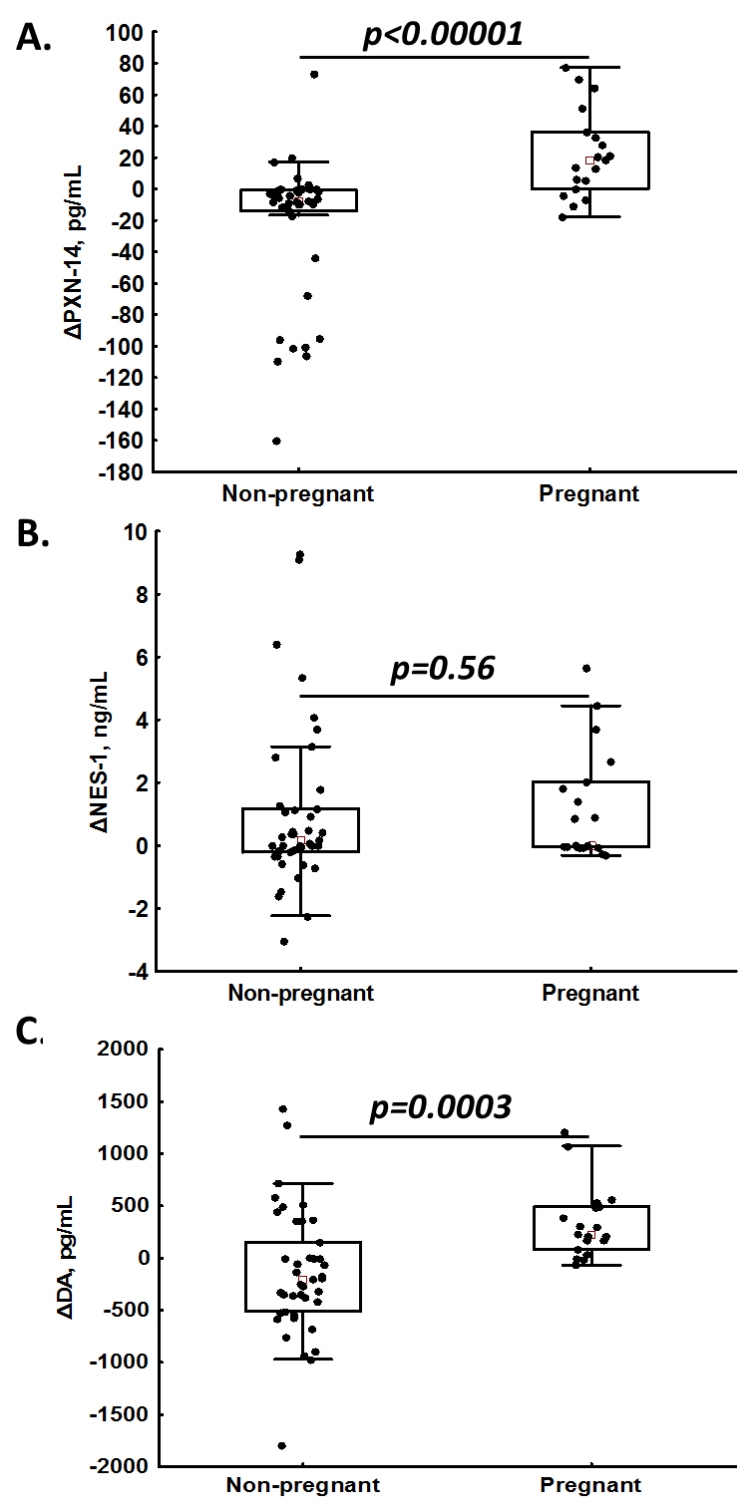
Intra-patient changes (Δ) in phoenixin-14 (PNX-14; panel (**A**)), nesfatin-1 (NES-1; panel (**B**)) and dopamine (DA; panel (**C**)) levels in non-pregnant and pregnant women.

In the univariable logistic regression analysis, PNX-14 (per 10 pg/mL, OR = 1.23, 95%CI 1.08–1.38) and DA (per 100 pg/mL, OR = 1.07, 95%CI 1.00–1.13) were associated with a positive pregnancy rate. In the multivariable logistic regression analysis, solely PNX-14 predicted pregnancy (per 10 pg/mL, OR = 1.23, 95%CI 1.07–1.39). In 83% (*n* = 14) of pregnant patients, we observed an increase in PNX-14 levels (delta > 0) after OS, while among non-pregnant women, increased PNX-14 was noted only in 13.2% (*n* = 5) of subjects (Figure 3A), giving a positive predictive value (PPV) of 74%. Of note, the positive predictive value (PPV) for NES-1 was 29% (increased post-treatment values were observed in 9/18 pregnant patients and 22/38 non-pregnant patients; Figure 3B), and for DA, the PPV was 57% (increased post-treatment values were observed in 16/18 pregnant patients and 12/38 non-pregnant patients; Figure 3C). 

## 4. Discussion

To our knowledge, this is the first study to show a higher basic OT level and post-OS PNX-14 concentration, which may influence fertilization and positive pregnancy rates. 

PNX-14 was discovered in 2013 as one of the neuropeptides that may influence ovarian cyclicity through the GnRH receptor [20]. PNX-14 enhances GnRH receptor expression in female pituitary cells increasing the GnRH effects, and it is involved in the preovulatory LH surge through the stimulation of GnRH [22]. There is compelling evidence that PNX-14 does not affect LH release or other hormones such as adrenocorticotropic hormone or prolactin itself [23]. Thus, PNX-14 may be one of the neuropeptides that sensitizes the pituitary to the action of GnRH [22]. Our findings showed higher post-treatment PNX-14 levels in women who achieved clinical pregnancy, which is in line with the above hypothesis. Despite that, OS influenced the release of reproductive hormones, namely, estradiol, LH and FSH, in a similar manner, and only one-third of patients achieved pregnancy. Importantly, this group was characterized by higher concentrations of PNX-14, NES-1 and DA, as well as markedly reduced OT. To evaluate the clinical impact of the above neuropeptides on pregnancy rates we broadened the analysis, which showed that among them, PNX-14 was a predictor of pregnancy after OS with the highest PPV for positive outcomes after OS. The current study revealed that PNX-14 had an impact on pregnancy rate after OS. Nevertheless, larger studies are needed to confirm our observation. 

On the other hand, our analysis revealed OT as a predictor of pregnancy before OS. This observation is in line with the previous studies showing that OT addition to the OS in anovulatory women increased the clinical pregnancy rates [12]. Interestingly, our study showed decreased post-treatment OT levels in a group that has experienced pregnancy following OS. In several studies, the efficacy of OT antagonists, i.e., atosiban for pregnancy outcomes in IVF, was analyzed; however, the results were contradictory revealing either increased [24,25,26] or no impact [27] of atosiban on pregnancy outcomes. Since OT is a neurohormone with known essential roles during labor, lactation, sexual behaviors and reproduction [28], our observation that OT may facilitate fertilization deserves further investigation. Moreover, increased OT levels were associated with elevated serum E_2_ concentrations, both under physiological and supraphysiological estrogenic conditions [29]. In our report, both serum E2 before and after OS treatment were higher in women who experienced pregnancy than in the group with negative pregnancy rates, which is consistent with the previous study [27]. We also demonstrated increased post-treatment DA levels in the pregnant group, which could be directly associated with an increase in GnRH pulsatility related to OS. Current studies have demonstrated the negative association between DA and GnRH release, suggesting that a reduction in the inhibitory effect of DA would have resulted in the rapid pulsatility of LH [15,30]. We found no associations between DA and LH, but DA weakly correlated with PRL, which may be explained by the small size of the population. Further studies with larger cohorts are needed to investigate whether OT and DA serum concentrations may have a positive effect on pregnancy rates after OS.

Moreover, our study showed elevated pre-treatment NES-1 levels in the pregnant compared to the non-pregnant group, suggesting that basic NES-1 may modify pregnancy rates after OS. Interestingly, OS abolished this effect, which may be associated with the observation that an OS-related increase in NES-1 levels was observed in half of pregnant women and the correlation between NES-1 and PNX-14 was negative. Our results are in line with the other study [31], where no relationship between the pregnancy rate and NES-1 levels was confirmed. Nevertheless, women with elevated intrafollicular fluid NES-1 concentration had a higher oocyte rate and good-quality embryos for cryopreservation [31]. On the other hand, Varlı et al. showed that higher serum NES-1 levels were observed in women with confirmed clinical pregnancy compared to the remainder [32]. 

Additionally, several experimental and clinical studies revealed either elevated [11,33,34,35] or decreased [35,36,37,38] NES-1 levels in PCOS. Nevertheless, nesfatin-1 injections reduced LH and GnRH expressions in the hypothalamus [39]. Therefore, it can be speculated that NES-1 could prevent the development of PCOS by its regulatory role on the HPO [40]. Despite it being suggested that NES-1 plays an important role in the ovarian function and reproduction [41,42,43], the NES-1 role in the HPO axis modulation is complicated [43,44], varying with different species and gender, and shows contradictory effects in vivo and in vitro [45]. Then, further investigations are needed to explain the role of NES-1 during fertilization and pregnancy.

To underline the important role of the neuropeptides in reproductive disorders and pregnancy outcomes, the position of another peptide—Kisspeptin (KP)—should be presented. KP plays a key role in the production of GnRH and, therefore, regulates gonadal function. Proper KP function conditions unaltered endometrial glands development and function in the uterus, as well as leading to follicle and oocyte development along with ovulation. Undisturbed KP performance is related to proper trophoblast invasion and embryonic development [46].

This study has some limitations. First, the sample size was small; however, it represented the typical population undergoing OS treatment. Moreover, the study was adequately powered to detect intergroup differences in neuropeptide concentrations. Secondly, the study was not randomized, so we were not able to compare neuropeptide levels in pregnant women followed by OS and natural ovulation. Thirdly, the study was focused on clinical confirmation of pregnancy, and the evolution of the pregnancy was not recorded, as well as the determination of neuropeptides at the early stage of pregnancy not being performed, and this issue is under investigation.

## 5. Conclusions

This study is the first to show that elevated OT before and PNX-14 after OS are associated with positive pregnancy rates. Our data support the concept that PNX-14 modulates the HPO axis, which may have clinically relevant consequences during OS. Therefore, a determination of PNX-14 and OT might be helpful during the treatment of ovulatory disorders and to predict treatment outcomes reflected by an increased pregnancy rate. Further studies on larger cohorts are needed to elucidate the impact of neuropeptides on the HPO axis during OS.

## Figures and Tables

**Table 1:** Basic and hormonal characteristics of study population.

	Whole Cohort (*n* = 56)	Pregnant (*n* = 18)	Non-Pregnant (*n* = 38)	*p*
Basic characteristic				
Age (years) [mean (SD)]	31.89 (4.59)	32.9 (5.1)	31.1 (4.3)	0.07
BMI (kg/m^2^) [mean (SD)]	24.53 (4.83)	25.63 (4.7)	24.04 (4.23)	0.09
Time of infertility (mo) [mean (SD)]	17.11 (4.34)	17.0 (4.23)	17.3 (4.7)	0.13
AMH (ng/mL) [mean (SD)]	4.9 (5.52)	4.32 (4.02)	5.16 (6.1)	0.02
Pre-stimulation				
Estradiol (pmol) [mean (SD)]	156.67 (88.92)	162.25 (86.68)	144.2 (95.25)	0.04
LH (IU/L) [mean (SD)]	8.99 (4.29)	7.59 (4.38)	9.61 (4.16)	0.03
FSH (IU/L) [mean (SD)]	5.4 (1.43)	5.31 (1.37)	5.43 (1.47)	0.06
Prolactin (ng/mL) [mean (SD)]	284.71 (120.65)	280.41 (94.0)	286.64 (131.96)	0.21
TSH (uIU/mL) [mean (SD)]	1.63 (0.65)	1.66 (0.64)	1.61 (0.66)	0.09
Post-stimulation				
Estradiol (pmol) [mean (SD)]	865.64 (602.29)	898.29 (653.27)	790.14 (473.92)	0.03
LH (IU/L) [mean (SD)]	17.87 (15.7)	16.96 (12.83)	18.26 (16.94)	0.17
FSH (IU/L) [mean (SD)]	6.55 (2.99)	7.2 (3.48)	6.26 (2.76)	0.21
Prolactin (ng/mL) [mean (SD)]	303.4 (142.47)	265.06 (101.16)	319.97 (155.3)	0.06
TSH (uIU/mL) [mean (SD)]	1.6 (0.62)	1.56 (0.58)	1.61 (0.64)	0.5

Data are shown as mean ± standard deviation, median (interquartile range) or number. Abbreviations: BMI, body mass index; LH, luteinizing hormone; FSH, follicle-stimulating hormone; TSH, thyroid stimulating hormone; AMH, anti-müllerian hormone.

**Table 2:** Comparison of serum neuropeptide levels between pregnant and non-pregnant women, before and after ovarian stimulation.

	Whole Cohort (*n* = 56)	Pregnant (*n* = 18)	Non-Pregnant (*n* = 38)	*p* *
Pre-stimulation				
PNX-14 (pg/mL) [median (Q1–Q3)]	141.7 (122.5–218.5)	141.4 (131.3–237.95)	142.01 (121.64–200.78)	0.46
NES-1 (ng/mL) [median (Q1–Q3)]	4.21 (2.74–5.17)	4.64 (3.86–5.69)	3.59 (2.56–4.86)	0.04
DA (pg/mL) [median (Q1–Q3)]	1918 (1037–2674)	1835.5 (1079.4–2463.7)	1916.15 (902.21–2338.8)	0.46
OT (pg/mL) [median (Q1–Q3)]	284 (105–399)	368.66 (197.04–491.96)	256.08 (70.66–387.12)	0.01
Post-stimulation				
PNX-14 (pg/mL) [median (Q1–Q3)]	146.3 (125.4–179.7)	162.3 (148.7–274.2)	138.2 (118.2–170.2)	<0.001
NES-1 (ng/mL) [median (Q1–Q3)]	4.86 (3.41–6.87)	5.37 (4.51–8.42)	4.26 (3.21–5.98)	0.01
DA (pg/mL) [median (Q1–Q3)]	1873 (949–2545)	2253.85 (1529.7–3024.5)	1548.7 (825.22–2345.1)	0.02
OT (pg/mL) [median (Q1–Q3)]	227 (92–412)	99.22 (65.33–270.18)	263.33 (165.26–415.34)	0.03

Data are shown as median (interquartile range) or number; * pregnant vs. non-pregnant. Abbreviations: PNX-14, phoenixin-14; NES-1, nesfatin-1; body mass index; DA, dopamine; OT, oxytocin.

## Data Availability

The data presented in this study are available on request from the corresponding author.
